# The enrichment ratio of atomic contacts in the crystal structure of isomeric, triply protonated, 4′-functionalized terpyridine cations with [ZnCl_4_]^2−^ as counter-ion

**DOI:** 10.1107/S2056989018016250

**Published:** 2018-11-30

**Authors:** Juan Granifo, Sebastián Suárez, Ricardo Baggio

**Affiliations:** a Departamento de Ciencias Químicas y Recursos Naturales, Facultad de Ingeniería y Ciencias, Universidad de La Frontera, Casilla 54-D, Temuco, Chile; b Departamento de Química Inorgánica, Analítica y Química, Física/INQUIMAE-CONICET, Facultad de Ciencias Exactas y Naturales, Universidad de Buenos Aires, Buenos Aires, Argentina; c Gerencia de Investigación y Aplicaciones, Centro Atómico Constituyentes, Comisión Nacional de Energía Atómica, Buenos Aires, Argentina

**Keywords:** crystal structure, terpyridine, Anion⋯π inter­actions, Hirshfeld surfaces

## Abstract

We report herein the synthesis, crystallographic analysis and a study of the non-covalent inter­actions observed in the new 4′-substituted terpyridine-based derivative bis­[4′-(isoquinolin-2-ium-4-yl)-4,2′:6′,4′′-terpyridine-1,1′′-diium] tris-[tetra­chlorido­zincate(II)], (C_24_H_19_N_4_)_2_[ZnCl_4_]_3_ or (44TPH_3_)_2_[ZnCl_4_]_3_, where (44TPH_3_)^3+^ is the triply protonated cation 4′-(isoquinolinium-4-yl)-4,2′:6′,4′′ terpyridinium. The compound is similar in its formulation to the recently reported 2,2′:6′,2′′ terpyridinium analogue {bis­[4′-(isoquinolin-2-ium-4-yl)-2,2′:6′,2′′-terpyridine-1,1′′-diium] tris­[tetra­chlorido­zincate(II)] monohydrate; Granifo *et al.* (2017 ▸). *Acta Cryst.* C**73**, 1121–1130}, although rather different and much simpler in its structural features, mainly in the number and type of non-covalent inter­actions present, as well as in the supra­molecular structure they define.

## Abstract 2

We report herein the synthesis, crystallographic analysis and a study of the non-covalent inter­actions observed in the new 4′-substituted terpyridine-based derivative bis­[4′-(isoquinolin-2-ium-4-yl)-4,2′:6′,4′′-terpyridine-1,1′′-diium] tris-[tetra­chlorido­zincate(II)]. The compound is similar in its formulation to the recently reported 2,2′:6′,2′′ terpyridinium analogue, although rather different and much simpler in its structural features, mainly in the number and type of non-covalent inter­actions present, as well as in the supra­molecular structure they define.

## Chemical context   

We have recently reported the use of the 4′-pyridyl-substituted terpyridine 4′-(isoquinolin-4-yl)-2,2′:6′,2′′-terpyridine (22TP) in the synthesis of the tetra­chlorido­zincate salt (22TPH_3_)_2_[ZnCl_4_]_3_·H_2_O (II) containing the triply protonated cation (22TPH_3_)^3+^ (Granifo *et al.*, 2017 ▸). The structural study of (II) demonstrates the concerted way in which a series of non-covalent inter­actions, viz. hydrogen bonding, anion–*π* and *π*–*π* stacking, participate in the crystal packing. The repulsive nature of the *π*–*π* inter­action between the triply protonated (22TPH_3_)^3+^ cations is counteracted by the [ZnCl_4_]^2−^ anions through abundant peripheral hydrogen bonding and anion–*π* inter­actions to the aromatic rings. A useful tool to highlight those contacts, which are statistically favored in a given structure, is the ***enrichment ratios approach*** (Jelsch *et al.*, 2014 ▸) based on the Hirshfeld surface, and whose application in the present case showed unexpectedly large enrichment ratios for the cationic C⋯N contacts in (II) as compared to those in the unprotonated base 22TP. This result was rationalized through the atomic and ring natural bond order charges (NBO), calculated by Maclagan and co-workers (Maclagan *et al.*, 2015 ▸) for a series of aromatic *N*-heterocyclic compounds. Concisely, in a protonated species, the hydrogen and nitro­gen in the N—H group carry an almost constant charge *q*, with an average of *q*(H) = 0.43 ± 0.01 and *q*(N) = −0.46 ± 0.1. The other atoms in the aromatic rings, C and H, receive the remaining positive charge, *i.e*. 0.57 ± 0.01 unit charge. A further remarkable result is that the *q*(N) values appear almost invariant when going from the neutral to the proton­ated base. Now, given that protonation leads to an increase on the positive charge in the C atoms and that the negative charge of the N atoms is almost invariant, a natural conclusion is that this ought to enrich the cationic C⋯N inter­actions. In an attempt to explore the effect of the position of the protonated N atoms on this type of inter­action, we decided to protonate the already known isomeric base 4′-(isoquinolin-4-yl)-4,2′:6′,4′′-terpyridine (44TP) (Granifo *et al.*, 2015 ▸) and to study the crystal structure of the new related compound (44TPH_3_)_2_[ZnCl_4_]_3_ (I).
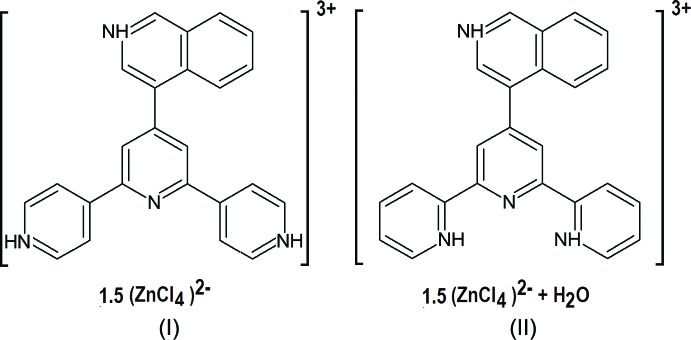


## Structural commentary   

**Figure 1:**
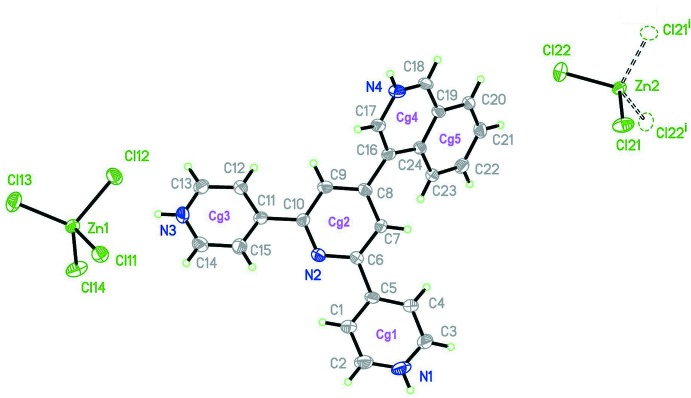
Mol­ecular view of the asymmetric unit in (I), with displacement ellipsoids drawn at the 50% probability level. Atom Zn2 lays onto a twofold symmetry axis. Symmetry code: (i) −*x* + 1, *y*, −*z* + 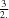

Fig. 1 ▸ shows the mol­ecular geometry as well as atom and ring labelling for (I). There is one (44TPH_3_)^3+^ independent cationic moiety, protonated at N1 and N3 in the lateral pyridine rings (hereinafter py) and at N4 in the iso­quinoline group (hereinafter, isq). The three negative charges required for charge balance are provided by one full independent [ZnCl_4_]^2−^ (tcz) anion in general position and a second one sitting on a twofold axis (thus providing only half of the charge). The general formulation is then (44TPH_3_)_2_[ZnCl_4_]_3_, similar to the 2,2′:6′,2′′ analogue (II) but without water as solvent. In this respect, the analogy goes a bit further: the pseudosymmetry observed in (II), which linked both (otherwise independent) (44TPH_3_)^3+^ cations becomes genuine symmetry in (I), expressed through the crystallographic twofold operation through the tcz group at Zn2.

Bond distances and angles are unremarkable in the (44TPH_3_)^3+^ moiety, with only minor departures from commonly accepted values in general, and from those in (II) in particular. The most relevant features come from the dihedral angles involving the inter­nal planar groups, and it is here where the mol­ecular differences with (II) are more apparent. The terpyridine nucleus presents significant out-of-plane rotations of the lateral pyridinium groups with regard to the central py one, and similarly with the pendant isq rings [dihedral angles: 2, 1 = 15.87 (16)°; 2, 3 = 25.80 (16)°; 2, 4 = 48.49 (15)°, plane labels taken from their N heteroatoms]. This large rotation of the isq group is required to avoid ‘bumping’ between the otherwise colliding atoms H7 and H23. The experimental *d*(H7⋯H23) distance is 2.36 Å, while in a perfectly planar disposition this value would collapse down to ≃ 0.80 Å. This ‘anti-bumping’ argument appears to be reinforced by the difference between the angles C16, [C24—C16—C8 = 124.7 (3)° > C17—C16—C8 = 116.2 (3)°], suggesting some kind of an H7⋯H23 repulsion.

## Supra­molecular features   

**Figure 2:**
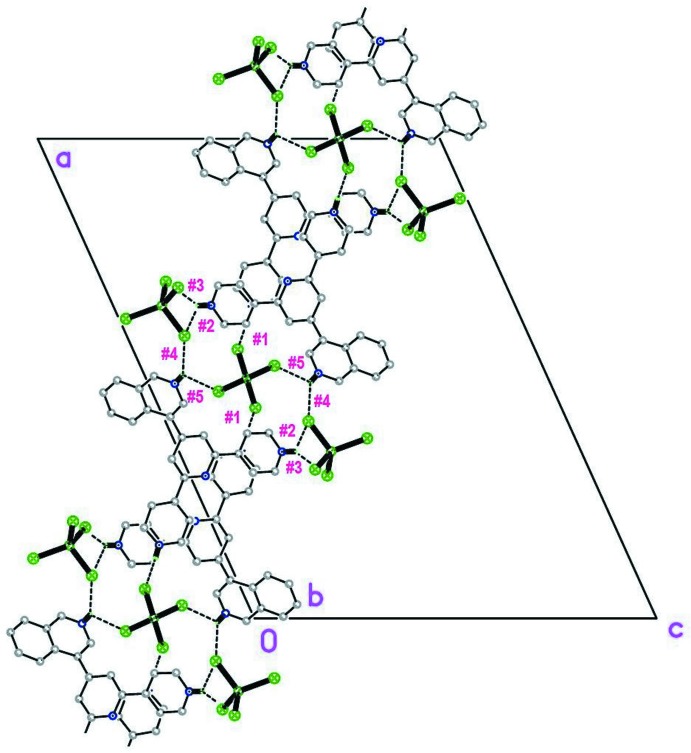
The broad (010) planar structure, shown sideways, along *b*.

**Figure 3:**
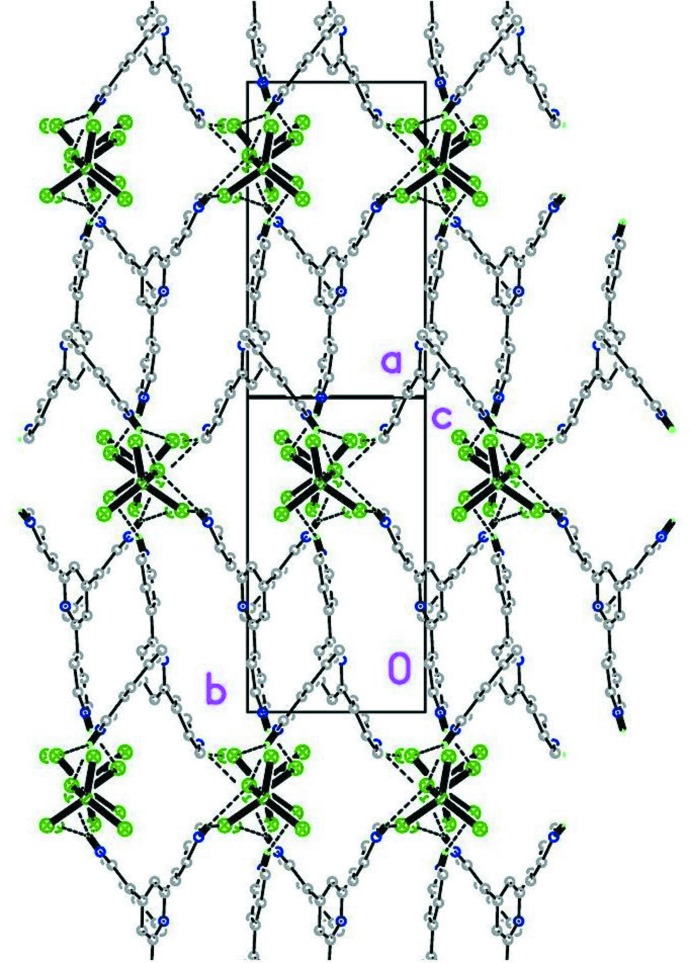
Same as Fig. 2 ▸, but shown along the plane normal, roughly [10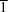].

**Figure 4:**
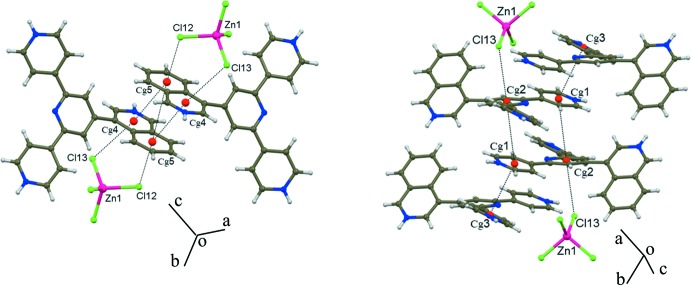
Anion⋯*π* inter­actions in (I) including the *π*-stacking involved.

As in (II), the most conspicuous aspect of the structure of (I) is its packing scheme, derived from a number of different inter­molecular inter­actions, presented in Table 1 ▸ (N/C—H⋯Cl), Table 2 ▸ (*π–π*) and Table 3 ▸ (Zn—Cl⋯π*/*π^*+*^), which for convenience of description have been assigned an individual ‘code’ or sequence number (from #1 to #17). Among these, hydrogen bonds are the most abundant and are clearly divided into two groups: stronger N—H⋯Cl (#1 to #5) and weaker C—H⋯Cl bonds (#6 to #10). Inter­actions #1 to #6 serve to link the (44TPH_3_)^3+^ cations to the [ZnCl_4_]^2−^ anions as shown in Fig. 2 ▸, to form broad 2D structures parallel to (10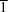) . Fig. 3 ▸, in turn, presents a view of these planar arrays along the plane normal. The remaining inter­actions (hydrogen bonds #7–#10, *π*–*π* contacts #11–#14 and Cl⋯*π* inter­actions #15–#17) link the superimposed planes roughly along [10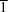], defining a well-connected 3D network. The so-called Cl⋯*π* inter­actions (Bauzá *et al.*, 2016 ▸; Gamez, 2014 ▸; Giese *et al.*, 2015 ▸, 2016 ▸) that involve the aromatic ring systems, either neutral *π* or charged π^*+*^, and the Cl^−^ anions are presented in Fig. 4 ▸.

## Hirshfeld surface and enrichment ratio   

**Figure 5:**
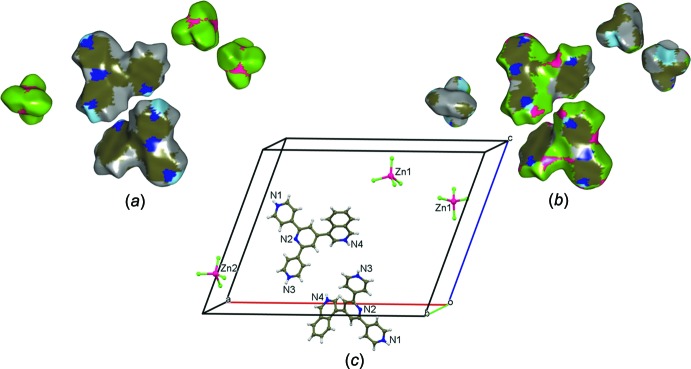
Right and left: Hirshfeld surfaces of the independent entities of (I) shown in (*c*) (conveniently set apart as to avoid overlapping) and colored in accordance with the species contributing most to the electron density on the surface; (*a*) surfaces coloured according to the inter­ior atoms (*b*) surfaces coloured according to the exterior atoms. Colour key: H_C_: grey, H_N_: light blue, C: dark brown, N: blue, Cl: green, Zn: purple.

**Figure 6:**
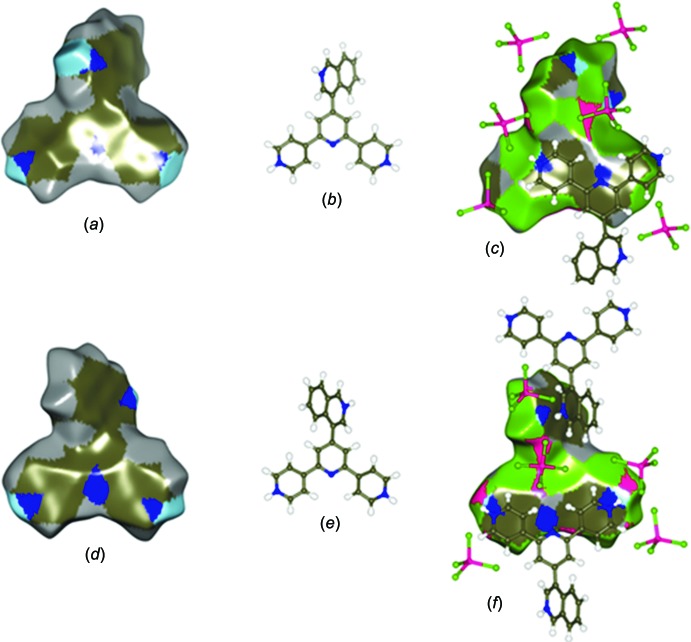
Hirshfeld surface of the (44TPH_3_)^3+^ cation coloured in accordance with the species contributing most to the electron density on the surface, showing (*a*)/(*c*) front and (*d*)/(*f*) back. In (*a*)/(*d*) the surface is coloured according to the inter­ior atoms and in (*c*)/(*f*) the surface is coloured according to the exterior atoms. The orientation of the structure inside the surface is shown in (*b*)/(*e*). Colour key: H_C_ grey, H_N_ light blue, C dark brown, N blue, Cl green and Zn magenta.

Calculations of the recently introduced enrichment ratio (ER) approach using the Hirshfeld surface methodology (Jelsch *et al.*, 2014 ▸) were performed with *MoProViewer* (Guillot *et al.*, 2014 ▸). Considering that the ER is the ratio between the actual contacts and those that should result from random ones, values larger than unity for any pair of elements mean they have a high tendency to form contacts in the crystal structure, the opposite happening for pairs with values lower than unity. The computed Hirshfeld surfaces and the corresponding contact ERs in the global structure of (I) are shown in Fig. 5 ▸ and Table 4 ▸, respectively. Since a tcz anion (Zn2) is located on a twofold axis, it was necessary to generate a dimer of the asymmetric unit in order to obtain the entire surface for each species (Fig. 5 ▸). As expected, the results show that the greatest contributions to the global surfaces (taking into account the inner and outer surfaces) are provided by C (27.56%), Cl (33.88%) and H_C_ (27.26%) atoms. On the other hand, visualization of the ERs discloses a remarkable increase in the C⋯N contacts in the (44TPH_3_)^3+^ cations (ER = 2.78; Table 4 ▸), as compared to those of neutral free 44TP (ER = 0.34; Table 5 ▸). As a way to specifically study the cation–cation inter­actions, the Hirshfeld surface and the respective ERs of the (44TPH_3_)^3+^ cation were computed. So, in Fig. 6 ▸, the coloured inter­ior/exterior Hirshfeld surfaces shows, as in the global situation, the relevance of the C (36.26%), Cl (25.08%) and H_C_ (27.34%) atoms, while values in Table 6 ▸ show that the C⋯N contacts are significantly enriched (ER = 2.15). When these results are compared with those obtained in (II), a very similar behavior is observed, *i.e*., in spite of changing the position of the protonated pyridyl N atoms, the system reorients itself as to favour the C⋯N inter­actions, evidencing the validity of the application of the criterion based on atomic charges.

## Database survey   

A search of the Cambridge Structural Database (CSD version 5.39, November 2017, update 3, May 2018; Groom *et al.*, 2016 ▸) for recently published structures with triply protonated (LH_3_)^3+^ cations showed just a handful of entries, viz: IRESII [2,4,6-tris­(2-pyridinio)pyridine trinitrate; Padhi *et al.*, 2011 ▸]; LEMVAC {2,2′-[4-(pyridinium-4-yl)pyridine-2,6-di­yl]dipyri­dinium trinitrate monohydrate; Seth *et al.*, 2013 ▸}; ODOHIA [tri­hydrogen 4′-(4-pyrid­yl)-2,2′:6′,2′′-terpyridine trinitrate bis­(nitric acid); Manna *et al.*, 2013 ▸]; LODHUJ [2,6-bis(pyridinium-2-yl)-4-(pyridinium-4-yl)pyridine tribromide trihydrate; Manna *et al.*, 2014*a* ▸]; FOTRUD [4′-(pyridinium-4-yl)-3,2′:6′,3′′-terpyridine-1,1′′-di-ium triperchlorate monohydrate; Manna *et al.*, 2014*b* ▸] and KEQYAJ {bis­[4′-(isoquin­olin-2-ium-4-yl)-2,2′:6′,2′′-terpyridine-1,1′′-diium] tris­[tetra­chlorido­zincate(II)] monohydrate (II); Granifo *et al.*, 2017 ▸}.

A characteristic found in these structures, in common with the case reported herein, is that only the N atoms of the three outermost pyridyl groups are protonated and that the lateral rings of the terpyridine portion adopt a *syn–syn* conformation with respect to the central pyridine ring. In most of the reported cases it was found that, in spite of the repulsive electrostatic nature between positively charged (LH_3_)^3+^ cations, the π–π stacking inter­actions appear enhanced when the π-system is charged. Due to lack of reported information, qu­anti­tative comparison of the ERs could only be made with the (already discussed) structure (II).

## Synthesis and crystallization   

The tetra­chlorido­zincate salt (44TPH_3_)_2_[ZnCl_4_]_3_ was prepared by the reaction of 4′-(isoquinolin-4-yl)-4,2′:6′,4′′- terpyridine (44TP; Granifo *et al.*, 2015 ▸), ZnCl_2_ and concentrated HCl (37%). 44TP (4.8 mg) was placed in a small beaker and dissolved with concentrated HCl (0.5 ml) and then 0.5 ml of water was added. To this solution was added an excess of ZnCl_2_ (48.0 mg) and the resulting solution was stirred for 1.5 min. The clear solution was allowed to stand at room temperature for a few days to give colourless block-shaped crystals, which were washed with methanol (3 × 1 ml) and then dried with hot air.

## Refinement   

Crystal data, data collection and structure refinement details are summarized in Table 7 ▸. H atoms were identified in an inter­mediate difference map, and treated differently in refinement: those attached to C were further idealized and finally allowed to ride with C—H = 0.93 Å, while those attached to N were refined with restrained N—H = 0.85 (1) Å. In all cases, H-atom displacement parameters were taken as *U*_iso_(H) =1.2 *U*_eq_(Host).

## Supplementary Material

Crystal structure: contains datablock(s) I, global. DOI: 10.1107/S2056989018016250/eb2014sup1.cif

Structure factors: contains datablock(s) I. DOI: 10.1107/S2056989018016250/eb2014Isup2.hkl

CCDC reference: 1879345

Additional supporting information:  crystallographic information; 3D view; checkCIF report

## Figures and Tables

**Table 1:** Hydrogen-bond geometry (Å, °) for (I)

Code	*D*—H⋯*A*	*D*—H	H⋯*A*	*D*⋯*A*	*D*—H⋯*A*
#1	N1—H1*N*⋯Cl21^i^	0.846 (10)	2.27 (3)	3.085 (4)	161 (3)
#2	N3—H3*N*⋯Cl12^ii^	0.846 (10)	2.71 (3)	3.291 (3)	127 (3)
#3	N3—H3*N*⋯Cl14^ii^	0.847 (10)	2.48 (4)	3.203 (4)	145 (3)
#4	N4—H4*N*⋯Cl12^iii^	0.850 (10)	2.41 (3)	3.125 (3)	142 (3)
#5	N4—H4*N*⋯Cl22^iv^	0.850 (10)	2.74 (3)	3.264 (3)	121 (3)
#6	C4—H4⋯Cl14^v^	0.93	2.72	3.316 (4)	123
#7	C7—H7⋯Cl14^v^	0.93	2.73	3.589 (3)	155
#8	C13—H13⋯Cl12^ii^	0.93	2.71	3.287 (4)	121
#9	C17—H17⋯Cl22^iv^	0.93	2.77	3.320 (3)	119
#10	C23—H23⋯Cl11^vii^	0.93	2.74	3.434 (4)	132

Symmetry codes: (i) −*x* + 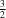, *y* + 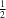, −*z* + 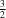; (ii) *x*, *y* + 1, *z*; (iii) −*x* + 1, *y*, −*z* + 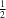; (iv) −*x* + 1, −*y* + 1, −*z* + 1; (v) *x*, −*y* + 1, *y* + 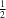; (vi) −*x* + 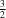, *y* + 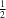, −*y* + 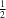; (vii) *x*, −*y* + 2, *y* + 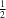.

**Table 2:** π–π contacts in (I) Ring codes as in Fig. 1 ▸. ccd: centroid-to-centroid distance; da: dihedral angle between rings; ipd: inter­planar distance, or (mean) distance from one plane to the neighbouring centroid. For details, see Janiak (2000 ▸)

Inter­action code	*Cg*⋯*Cg*	ccd (Å)	da (°)	ipd (Å)
#11	*Cg*4⋯*Cg*5^vii^	3.495 (2)	18.1 (15)	3.32 (2)
#12	*Cg*5⋯*Cg*5^vii^	3.843 (2)	0.0	3.35 (2)
#13	*Cg*1⋯*Cg*3^viii^	3.784 (2)	16.2 (14)	3.66 (2)
#14	*Cg*1⋯*Cg*2^ix^	4.149 (2)	15.9 (16)	3.85 (2)

Symmetry codes: (vii) −*x* + 1, −*y* + 2, −*z* + 1; (viii) −*x* + 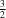, −*y* + 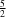, −*z* + 1; (ix) −*x* + 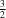, −*y* + 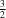, −*z* + 1.

**Table 3:** Anion⋯π and anion⋯π^+^ inter­actions (Å, °) in (I) *d* is the Cl⋯*X* distance where *X* is the atom in the ring nearest the Cl anion; α is the angle subtended by the Cl–*Cg* vector to the ring normal; β is the angle subtended by the *X*–*Cg* and *X*–*Cg* vectors (for β < 90°, the anion projects within the ring and for 90° < β, the anion projects outside the ring; *n* (in η^*n*^) is the number of inter­acting atoms. NB according to standard requirements for anion⋯π inter­actions (Giese *et al.* 2015 ▸, 2016 ▸), β should be < 100°.

Code	Zn—Cl⋯*Cg*	Cl⋯*Cg*	*d*	*α*	*β*	η^*n*^
#15	Zn1—Cl12⋯*Cg*5^*x*^	3.739 (2)	3.671	14.5	82.0	*η^2^*
#16	Zn1—Cl13⋯*Cg*2^xi^	3.760 (2)	3.829	8.60	76.5	*η^1^*
#17	Zn1—Cl13⋯*Cg*4^xii^	4.084 (2)	3.748	23.9	94.3	*η^1^*

Symmetry codes: (*x*) *x*, −*y* + 1, *z* − 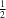; (xi) *x*, −*y* + 2, *z* − 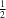; (xii) *x*, −*y* + 1, *z* − 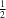.

**Table 4:** Hirshfeld contact surfaces and ERs for (44TPH_3_)_2_[ZnCl_4_]_3_ computed around the two (44TPH_3_)^3+^ cations and the three [ZnCl_4_]^2−^ anions The H atoms bound to carbon (H_C_) and nitro­gen (H_N_) are differentiated. The first column corresponds to ‘inter­ior’ atoms and the remaining columns to ‘exterior’ ones.

	C	N	Cl	Zn	H_C_	H_N_
Surface inter­ior (%)	28.66	2.65	33.36	3.49	26.38	5.46
Surface exterior (%)	26.45	2.20	34.40	3.01	28.15	5.80
						
Enrichment ratios (reciprocal contacts merged)						
C	1.74					
N	2.78	0.24				
Cl	0.89	0.21	0.27			
Zn	0.92	0.00	0.10	0.00		
H_C_	0.35	0.59	1.91	2.33	0.60	
H_N_	0.49	0.00	2.36	1.40	0.07	0.00

**Table 5:** Hirshfeld contact surfaces and ERs for 44TP The first column corresponds to ‘inter­ior’ atoms and the remaining columns to ‘exterior’ ones.

	C	N	H_C_
Surface inter­ior (%)	42.44	10.43	47.13
Surface exterior (%)	40.81	10.44	48.74
			
Enrichment ratios (reciprocal contacts merged)			
C	1.28		
N	0.34	0.44	
H_C_	0.90	1.69	0.94

**Table 6:** Hirshfeld contact surfaces and ERs for (44TPH_3_)^3+^ The H atoms bound to carbon (H_C_) and nitro­gen (H_N_) are differentiated. The first column corresponds to ‘inter­ior’ atoms and the remaining columns to ‘exterior’ ones.

	C	N	Cl	Zn	H_C_	H_N_
Surface inter­ior (%)	44.29	4.09	0.00	0.00	42.66	8.96
Surface exterior (%)	28.22	3.60	50.16	4.66	12.03	1.34
						
Enrichment ratios (reciprocal contacts merged)						
C	1.62					
N	2.15	0.43				
Cl	0.62	0.16	NaN			
Zn	0.76	0.00	NaN	NaN		
H_C_	0.49	0.65	1.34	1.37	1.29	
H_N_	0.73	0.00	1.64	0.88	0.18	0.00

**Table 7:** Experimental details

Crystal data	
Chemical formula	(C_24_H_19_N_4_)_2_[ZnCl_4_]_3_
*M*_r_	1348.37
Crystal system, space group	Monoclinic, *C*2/*c*
Temperature (K)	295
*a*, *b*, *c* (Å)	30.642 (2), 8.0866 (4), 23.413 (2)
β (°)	114.316 (7)
*V* (Å^3^)	5286.8 (7)
*Z*	4
Radiation type	Mo *K*α
μ (mm^−1^)	2.00
Crystal size (mm)	0.34 × 0.20 × 0.14

Data collection	
Diffractometer	Oxford Diffraction Xcalibur, Sapphire3
Absorption correction	Multi-scan (*CrysAlis PRO*; Rigaku OD, 2015 ▸)
*T*_min_, *T*_max_	0.58, 0.82
No. of measured, independent and observed [*I* > 2σ(*I*)] reflections	23819, 6416, 4482
*R*_int_	0.065
(sin θ/λ)_max_ (Å^−1^)	0.690

Refinement	
*R*[*F* ^2^ > 2σ(*F* ^2^)], *wR*(*F* ^2^), *S*	0.045, 0.106, 1.08
No. of reflections	6416
No. of parameters	330
No. of restraints	3
H-atom treatment	H atoms treated by a mixture of independent and constrained refinement
Δρ_max_, Δρ_min_ (e Å^−3^)	0.62, −0.60

Computer programs: *CrysAlis PRO* (Rigaku OD, 2015 ▸), *SHELXS97* and *SHELXTL* (Sheldrick, 2008 ▸), *SHELXL2014* (Sheldrick, 2015 ▸)and *PLATON* (Spek, 2009 ▸).

## supplementary crystallographic information

### Crystal data

**Table:**

(C_24_H_19_N_4_)_2_[ZnCl_4_]_3_	*F*(000) = 2704
*M**_r_* = 1348.37	*D*_x_ = 1.694 Mg m^−^^3^
Monoclinic, *C*2/*c*	Mo *K*α radiation, λ = 0.71073 Å
*a* = 30.642 (2) Å	Cell parameters from 4699 reflections
*b* = 8.0866 (4) Å	θ = 3.7–27.5°
*c* = 23.413 (2) Å	µ = 2.00 mm^−^^1^
β = 114.316 (7)°	*T* = 295 K
*V* = 5286.8 (7) Å^3^	Blocks, colourless
*Z* = 4	0.34 × 0.20 × 0.14 mm

### Data collection

**Table:**

Oxford Diffraction Xcalibur, Sapphire3 diffractometer	6416 independent reflections
Radiation source: fine-focus sealed X-ray tube, Enhance (Mo) X-ray Source	4482 reflections with *I* > 2σ(*I*)
Graphite monochromator	*R*_int_ = 0.065
ω scans	θ_max_ = 29.4°, θ_min_ = 3.7°
Absorption correction: multi-scan (CrysAlisPro; Rigaku OD, 2015)	*h* = −39→41
*T*_min_ = 0.58, *T*_max_ = 0.82	*k* = −10→10
23819 measured reflections	*l* = −30→31

### Refinement

**Table:**

Refinement on *F*^2^	3 restraints
Least-squares matrix: full	Hydrogen site location: mixed
*R*[*F*^2^ > 2σ(*F*^2^)] = 0.045	H atoms treated by a mixture of independent and constrained refinement
*wR*(*F*^2^) = 0.106	*w* = 1/[σ^2^(*F*_o_^2^) + (0.0313*P*)^2^ + 4.8559*P*] where *P* = (*F*_o_^2^ + 2*F*_c_^2^)/3
*S* = 1.08	(Δ/σ)_max_ = 0.002
6416 reflections	Δρ_max_ = 0.62 e Å^−^^3^
330 parameters	Δρ_min_ = −0.60 e Å^−^^3^

### Special details

**Table:**

Geometry. All esds (except the esd in the dihedral angle between two l.s. planes) are estimated using the full covariance matrix. The cell esds are taken into account individually in the estimation of esds in distances, angles and torsion angles; correlations between esds in cell parameters are only used when they are defined by crystal symmetry. An approximate (isotropic) treatment of cell esds is used for estimating esds involving l.s. planes.

### Fractional atomic coordinates and isotropic or equivalent isotropic displacement parameters (Å^2^)

**Table:**

	*x*	*y*	*z*	*U*_iso_*/*U*_eq_	
Zn1	0.65199 (2)	0.59124 (5)	0.12147 (2)	0.02480 (12)	
Cl11	0.68969 (3)	0.80880 (11)	0.18160 (5)	0.0407 (3)	
Cl12	0.58917 (3)	0.50894 (12)	0.14395 (5)	0.0419 (3)	
Cl13	0.62471 (4)	0.63226 (13)	0.01753 (4)	0.0418 (2)	
Cl14	0.70191 (3)	0.37027 (10)	0.15801 (4)	0.0325 (2)	
Zn2	0.500000	0.40642 (7)	0.750000	0.02449 (14)	
Cl21	0.56071 (3)	0.22938 (11)	0.76067 (5)	0.0384 (2)	
Cl22	0.47311 (3)	0.57574 (10)	0.66606 (4)	0.0342 (2)	
N1	0.84733 (11)	0.9147 (4)	0.65467 (16)	0.0383 (8)	
H1N	0.8754 (6)	0.887 (4)	0.6794 (16)	0.046*	
N2	0.70304 (9)	1.0267 (3)	0.46155 (13)	0.0257 (6)	
N3	0.65260 (11)	1.2242 (4)	0.24416 (14)	0.0327 (7)	
H3N	0.6521 (13)	1.273 (4)	0.2119 (11)	0.039*	
N4	0.51052 (10)	0.6700 (3)	0.43396 (14)	0.0281 (7)	
H4N	0.4922 (11)	0.609 (3)	0.4043 (12)	0.034*	
C1	0.79534 (11)	0.9775 (4)	0.55141 (17)	0.0328 (9)	
H1	0.790536	1.005648	0.510738	0.039*	
C2	0.84084 (12)	0.9559 (5)	0.59660 (19)	0.0380 (9)	
H2	0.867084	0.969995	0.586770	0.046*	
C3	0.81105 (13)	0.9004 (4)	0.67238 (18)	0.0377 (10)	
H3	0.816987	0.874321	0.713614	0.045*	
C4	0.76494 (12)	0.9249 (4)	0.62865 (17)	0.0334 (9)	
H4	0.739506	0.919950	0.640532	0.040*	
C5	0.75647 (11)	0.9572 (4)	0.56675 (16)	0.0257 (8)	
C6	0.70667 (11)	0.9661 (4)	0.51656 (15)	0.0233 (7)	
C7	0.66782 (11)	0.9041 (4)	0.52621 (15)	0.0228 (7)	
H7	0.672074	0.860642	0.564933	0.027*	
C8	0.62267 (11)	0.9081 (4)	0.47714 (16)	0.0221 (7)	
C9	0.61866 (11)	0.9763 (4)	0.42063 (16)	0.0251 (7)	
H9	0.588853	0.984287	0.387052	0.030*	
C10	0.65932 (11)	1.0322 (4)	0.41467 (15)	0.0230 (7)	
C11	0.65678 (11)	1.0990 (4)	0.35449 (15)	0.0232 (7)	
C12	0.61460 (12)	1.1688 (4)	0.31040 (16)	0.0285 (8)	
H12	0.587272	1.172068	0.318353	0.034*	
C13	0.61339 (13)	1.2323 (4)	0.25572 (17)	0.0313 (8)	
H13	0.585546	1.280842	0.226775	0.038*	
C14	0.69363 (13)	1.1557 (5)	0.28393 (18)	0.0366 (9)	
H14	0.719948	1.150150	0.273842	0.044*	
C15	0.69625 (13)	1.0939 (4)	0.33971 (17)	0.0321 (8)	
H15	0.724803	1.048051	0.368031	0.039*	
C16	0.57945 (11)	0.8377 (4)	0.48229 (15)	0.0225 (7)	
C17	0.55171 (11)	0.7358 (4)	0.43484 (16)	0.0270 (8)	
H17	0.561020	0.710850	0.402698	0.032*	
C18	0.49493 (11)	0.7020 (4)	0.47743 (16)	0.0253 (8)	
H18	0.466264	0.655910	0.474464	0.030*	
C19	0.52111 (11)	0.8044 (4)	0.52799 (16)	0.0236 (7)	
C20	0.50381 (11)	0.8408 (4)	0.57389 (16)	0.0262 (8)	
H20	0.475249	0.794643	0.571249	0.031*	
C21	0.52914 (12)	0.9435 (4)	0.62180 (16)	0.0301 (8)	
H21	0.518261	0.966419	0.652638	0.036*	
C22	0.57181 (12)	1.0155 (4)	0.62503 (16)	0.0283 (8)	
H22	0.588456	1.087141	0.657908	0.034*	
C23	0.58967 (11)	0.9837 (4)	0.58143 (15)	0.0243 (7)	
H23	0.617993	1.033407	0.584754	0.029*	
C24	0.56479 (11)	0.8747 (4)	0.53117 (15)	0.0218 (7)	

### Atomic displacement parameters (Å^2^)

**Table:**

	*U*^11^	*U*^22^	*U*^33^	*U*^12^	*U*^13^	*U*^23^
Zn1	0.0216 (2)	0.0276 (2)	0.0238 (2)	0.00225 (15)	0.00800 (17)	0.00170 (16)
Cl11	0.0324 (5)	0.0322 (5)	0.0413 (6)	0.0011 (4)	−0.0012 (4)	−0.0048 (4)
Cl12	0.0251 (5)	0.0506 (6)	0.0529 (6)	0.0114 (4)	0.0190 (4)	0.0275 (5)
Cl13	0.0453 (6)	0.0558 (6)	0.0245 (5)	0.0109 (5)	0.0146 (4)	0.0042 (4)
Cl14	0.0280 (4)	0.0315 (5)	0.0396 (5)	0.0075 (3)	0.0156 (4)	0.0052 (4)
Zn2	0.0206 (3)	0.0297 (3)	0.0206 (3)	0.000	0.0060 (2)	0.000
Cl21	0.0251 (5)	0.0401 (5)	0.0431 (6)	0.0051 (4)	0.0071 (4)	−0.0101 (4)
Cl22	0.0450 (5)	0.0337 (5)	0.0243 (5)	0.0047 (4)	0.0147 (4)	0.0039 (4)
N1	0.0213 (16)	0.0404 (19)	0.040 (2)	0.0015 (14)	−0.0010 (15)	0.0030 (16)
N2	0.0209 (14)	0.0291 (15)	0.0278 (17)	−0.0009 (12)	0.0107 (12)	0.0032 (13)
N3	0.0368 (18)	0.0399 (18)	0.0223 (17)	−0.0082 (14)	0.0132 (14)	0.0010 (14)
N4	0.0220 (15)	0.0294 (16)	0.0305 (18)	−0.0063 (12)	0.0084 (13)	−0.0068 (13)
C1	0.0209 (18)	0.045 (2)	0.031 (2)	−0.0024 (16)	0.0089 (15)	0.0016 (17)
C2	0.0211 (18)	0.050 (2)	0.039 (2)	0.0001 (16)	0.0088 (17)	−0.0028 (19)
C3	0.033 (2)	0.040 (2)	0.030 (2)	−0.0108 (17)	0.0027 (17)	0.0060 (17)
C4	0.0229 (18)	0.044 (2)	0.028 (2)	−0.0078 (15)	0.0058 (15)	0.0030 (17)
C5	0.0185 (16)	0.0262 (17)	0.030 (2)	−0.0052 (13)	0.0075 (14)	0.0007 (15)
C6	0.0211 (17)	0.0260 (17)	0.0235 (18)	−0.0026 (14)	0.0097 (14)	0.0004 (14)
C7	0.0221 (17)	0.0276 (17)	0.0182 (17)	−0.0003 (13)	0.0079 (13)	0.0005 (14)
C8	0.0210 (16)	0.0231 (16)	0.0243 (18)	−0.0029 (13)	0.0115 (14)	−0.0044 (14)
C9	0.0181 (16)	0.0290 (18)	0.0266 (19)	0.0004 (13)	0.0077 (14)	−0.0016 (15)
C10	0.0191 (16)	0.0286 (17)	0.0208 (18)	0.0022 (13)	0.0078 (13)	0.0013 (14)
C11	0.0196 (16)	0.0270 (17)	0.0224 (18)	−0.0011 (13)	0.0081 (14)	−0.0032 (14)
C12	0.0246 (18)	0.0316 (19)	0.029 (2)	−0.0014 (14)	0.0109 (15)	0.0060 (16)
C13	0.029 (2)	0.0311 (19)	0.028 (2)	−0.0032 (15)	0.0059 (16)	0.0028 (16)
C14	0.028 (2)	0.052 (2)	0.033 (2)	−0.0050 (17)	0.0166 (17)	−0.0026 (18)
C15	0.0273 (19)	0.041 (2)	0.029 (2)	0.0041 (16)	0.0129 (16)	0.0056 (17)
C16	0.0168 (16)	0.0243 (17)	0.0235 (18)	0.0017 (13)	0.0053 (13)	0.0014 (14)
C17	0.0194 (17)	0.0365 (19)	0.0259 (19)	0.0008 (14)	0.0101 (14)	0.0000 (15)
C18	0.0175 (16)	0.0268 (18)	0.031 (2)	−0.0029 (13)	0.0093 (14)	0.0032 (15)
C19	0.0172 (16)	0.0250 (17)	0.0270 (19)	0.0016 (13)	0.0075 (14)	0.0060 (14)
C20	0.0198 (17)	0.0330 (19)	0.0262 (19)	0.0012 (14)	0.0099 (14)	0.0091 (15)
C21	0.0289 (19)	0.040 (2)	0.026 (2)	0.0048 (16)	0.0157 (16)	0.0060 (16)
C22	0.0294 (19)	0.0300 (19)	0.0220 (18)	−0.0021 (15)	0.0071 (15)	−0.0008 (15)
C23	0.0221 (17)	0.0263 (17)	0.0233 (18)	−0.0029 (13)	0.0080 (14)	0.0025 (14)
C24	0.0183 (16)	0.0226 (16)	0.0247 (18)	0.0029 (13)	0.0091 (13)	0.0052 (14)

### Geometric parameters (Å, º)

**Table:**

Zn1—Cl13	2.2485 (10)	C7—H7	0.9300
Zn1—Cl11	2.2521 (10)	C8—C9	1.391 (5)
Zn1—Cl14	2.2775 (9)	C8—C16	1.491 (4)
Zn1—Cl12	2.2935 (10)	C9—C10	1.385 (4)
Zn2—Cl22	2.2545 (9)	C9—H9	0.9300
Zn2—Cl22^i^	2.2545 (9)	C10—C11	1.481 (5)
Zn2—Cl21	2.2782 (9)	C11—C15	1.389 (5)
Zn2—Cl21^i^	2.2783 (9)	C11—C12	1.399 (4)
N1—C2	1.333 (5)	C12—C13	1.366 (5)
N1—C3	1.342 (5)	C12—H12	0.9300
N1—H1N	0.846 (10)	C13—H13	0.9300
N2—C10	1.338 (4)	C14—C15	1.369 (5)
N2—C6	1.339 (4)	C14—H14	0.9300
N3—C13	1.337 (5)	C15—H15	0.9300
N3—C14	1.338 (5)	C16—C17	1.363 (4)
N3—H3N	0.847 (10)	C16—C24	1.423 (5)
N4—C18	1.316 (4)	C17—H17	0.9300
N4—C17	1.362 (4)	C18—C19	1.396 (5)
N4—H4N	0.850 (10)	C18—H18	0.9300
C1—C2	1.371 (5)	C19—C20	1.411 (5)
C1—C5	1.388 (5)	C19—C24	1.427 (4)
C1—H1	0.9300	C20—C21	1.354 (5)
C2—H2	0.9300	C20—H20	0.9300
C3—C4	1.376 (5)	C21—C22	1.405 (5)
C3—H3	0.9300	C21—H21	0.9300
C4—C5	1.388 (5)	C22—C23	1.367 (5)
C4—H4	0.9300	C22—H22	0.9300
C5—C6	1.496 (4)	C23—C24	1.416 (4)
C6—C7	1.393 (4)	C23—H23	0.9300
C7—C8	1.388 (4)		
			
Cl13—Zn1—Cl11	115.32 (4)	C10—C9—H9	120.2
Cl13—Zn1—Cl14	114.53 (4)	C8—C9—H9	120.2
Cl11—Zn1—Cl14	106.61 (4)	N2—C10—C9	123.0 (3)
Cl13—Zn1—Cl12	108.54 (4)	N2—C10—C11	115.8 (3)
Cl11—Zn1—Cl12	110.25 (4)	C9—C10—C11	121.2 (3)
Cl14—Zn1—Cl12	100.58 (4)	C15—C11—C12	117.6 (3)
Cl22—Zn2—Cl22^i^	105.21 (5)	C15—C11—C10	121.1 (3)
Cl22—Zn2—Cl21	117.78 (4)	C12—C11—C10	121.3 (3)
Cl22^i^—Zn2—Cl21	107.29 (3)	C13—C12—C11	120.3 (3)
Cl22—Zn2—Cl21^i^	107.30 (3)	C13—C12—H12	119.9
Cl22^i^—Zn2—Cl21^i^	117.77 (4)	C11—C12—H12	119.9
Cl21—Zn2—Cl21^i^	102.14 (5)	N3—C13—C12	119.4 (3)
C2—N1—C3	122.8 (3)	N3—C13—H13	120.3
C2—N1—H1N	117 (3)	C12—C13—H13	120.3
C3—N1—H1N	120 (3)	N3—C14—C15	118.9 (4)
C10—N2—C6	117.4 (3)	N3—C14—H14	120.5
C13—N3—C14	123.0 (3)	C15—C14—H14	120.5
C13—N3—H3N	118 (3)	C14—C15—C11	120.8 (3)
C14—N3—H3N	119 (3)	C14—C15—H15	119.6
C18—N4—C17	122.8 (3)	C11—C15—H15	119.6
C18—N4—H4N	115 (3)	C17—C16—C24	119.0 (3)
C17—N4—H4N	122 (3)	C17—C16—C8	116.2 (3)
C2—C1—C5	119.6 (4)	C24—C16—C8	124.7 (3)
C2—C1—H1	120.2	N4—C17—C16	120.7 (3)
C5—C1—H1	120.2	N4—C17—H17	119.6
N1—C2—C1	119.7 (4)	C16—C17—H17	119.6
N1—C2—H2	120.1	N4—C18—C19	120.5 (3)
C1—C2—H2	120.1	N4—C18—H18	119.8
N1—C3—C4	119.0 (4)	C19—C18—H18	119.8
N1—C3—H3	120.5	C18—C19—C20	120.3 (3)
C4—C3—H3	120.5	C18—C19—C24	118.6 (3)
C3—C4—C5	119.8 (4)	C20—C19—C24	121.0 (3)
C3—C4—H4	120.1	C21—C20—C19	119.5 (3)
C5—C4—H4	120.1	C21—C20—H20	120.3
C1—C5—C4	118.8 (3)	C19—C20—H20	120.3
C1—C5—C6	119.8 (3)	C20—C21—C22	120.1 (3)
C4—C5—C6	121.4 (3)	C20—C21—H21	119.9
N2—C6—C7	123.3 (3)	C22—C21—H21	119.9
N2—C6—C5	115.3 (3)	C23—C22—C21	122.1 (3)
C7—C6—C5	121.2 (3)	C23—C22—H22	118.9
C8—C7—C6	119.0 (3)	C21—C22—H22	118.9
C8—C7—H7	120.5	C22—C23—C24	119.6 (3)
C6—C7—H7	120.5	C22—C23—H23	120.2
C7—C8—C9	117.7 (3)	C24—C23—H23	120.2
C7—C8—C16	122.7 (3)	C23—C24—C16	124.0 (3)
C9—C8—C16	119.6 (3)	C23—C24—C19	117.6 (3)
C10—C9—C8	119.6 (3)	C16—C24—C19	118.3 (3)

Symmetry code: (i) −*x*+1, *y*, −*z*+3/2.

### Hydrogen-bond geometry (Å, º)

**Table:**

*D*—H···*A*	*D*—H	H···*A*	*D*···*A*	*D*—H···*A*
N1—H1*N*···Cl21^ii^	0.85 (1)	2.27 (2)	3.084 (3)	161 (3)
N3—H3*N*···Cl12^iii^	0.85 (1)	2.71 (3)	3.291 (3)	127 (3)
N3—H3*N*···Cl14^iii^	0.85 (1)	2.47 (2)	3.203 (3)	144 (3)
N4—H4*N*···Cl12^iv^	0.85 (1)	2.41 (2)	3.125 (3)	142 (3)
N4—H4*N*···Cl22^v^	0.85 (1)	2.74 (3)	3.264 (3)	121 (3)
C2—H2···Cl13^vi^	0.93	2.87	3.551 (4)	131
C3—H3···Cl11^vii^	0.93	2.94	3.823 (4)	158
C4—H4···Cl14^viii^	0.93	2.72	3.316 (4)	123
C7—H7···Cl14^viii^	0.93	2.73	3.589 (3)	155
C12—H12···Cl22^ix^	0.93	2.88	3.607 (4)	136
C13—H13···Cl12^iii^	0.93	2.71	3.287 (4)	121
C14—H14···Cl11^vi^	0.93	2.83	3.548 (4)	135
C15—H15···Cl14^vi^	0.93	2.93	3.588 (4)	129
C17—H17···Cl22^v^	0.93	2.77	3.320 (4)	119
C18—H18···Cl12^iv^	0.93	2.85	3.335 (3)	114
C18—H18···Cl13^iv^	0.93	2.88	3.759 (3)	159

Symmetry codes: (ii) −*x*+3/2, *y*+1/2, −*z*+3/2; (iii) *x*, *y*+1, *z*; (iv) −*x*+1, *y*, −*z*+1/2; (v) −*x*+1, −*y*+1, −*z*+1; (vi) −*x*+3/2, *y*+1/2, −*z*+1/2; (vii) −*x*+3/2, −*y*+3/2, −*z*+1; (viii) *x*, −*y*+1, *z*+1/2; (ix) −*x*+1, −*y*+2, −*z*+1.
